# Direct cholangioscopic visualization during placement of overlapping stents in malignant duodenal horizontal segment obstruction

**DOI:** 10.1055/a-2794-7617

**Published:** 2026-02-24

**Authors:** Junzhen Li, Chumei Huang, Mufeng Ye, Yutao Zhao, Weiwen Shi, Jian Qi, Man Yang

**Affiliations:** 1 543160 Department of Gastroenterology and Hepatology, Digestive Medicine Center, The Seventh Affiliated Hospital, Sun Yat-Sen University, Shenzhen, China

**Fig. 1:**
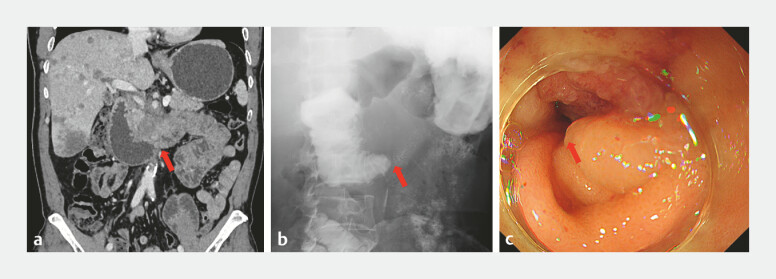
Luminal stenosis at the horizontal portion of duodenum (red arrow), due to the invasion of pancreatic carcinoma. **a** An abdominal computed tomography scan view. **b** An upper gastrointestinal series view. **c** An endoscopy view.

A 51-year-old man was admitted for nausea and vomiting for 2 months. He was diagnosed with pancreatic cancer with liver, lung, and bone metastasis 3 months ago and had completed four cycles of chemotherapy. An abdominal computed tomography scan revealed pancreatic head carcinoma with duodenal involvement, indicating malignant obstruction ( Fig. 1 **a** ). Upper gastrointestinal series demonstrated luminal stenosis at the horizontal portion of duodenum, with dilation of the descending duodenum ( Fig. 1 **b** ).

**Fig. 2:**
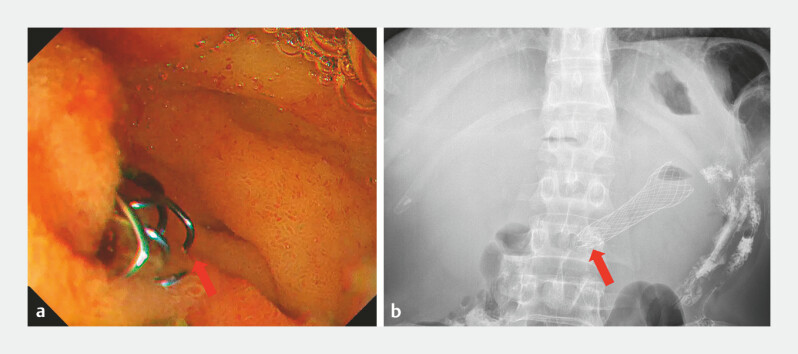
The proximal end of the stent (red arrow) failed to achieve complete expansion. **a** An endoscopy view and **b** an abdominal X-ray view.

Endoscopy identified a stricture at the duodenal horizontal segment ( Fig. 1 **c** ). A 25 × 60 mm uncovered duodenal stent was deployed across the stenotic segment ( Fig. 2 **a** ). An abdominal X-ray showed that the proximal end of the stent failed to achieve well expansion due to its deep deployment and the angulation at the duodenal horizontal segment ( Fig. 2 **b** ), with retained food contents in the dilated stomach and descending duodenum. An ultrathin endoscope could not be advanced through the stent due to the angulation at the duodenal horizontal segment.

**Fig. 3:**
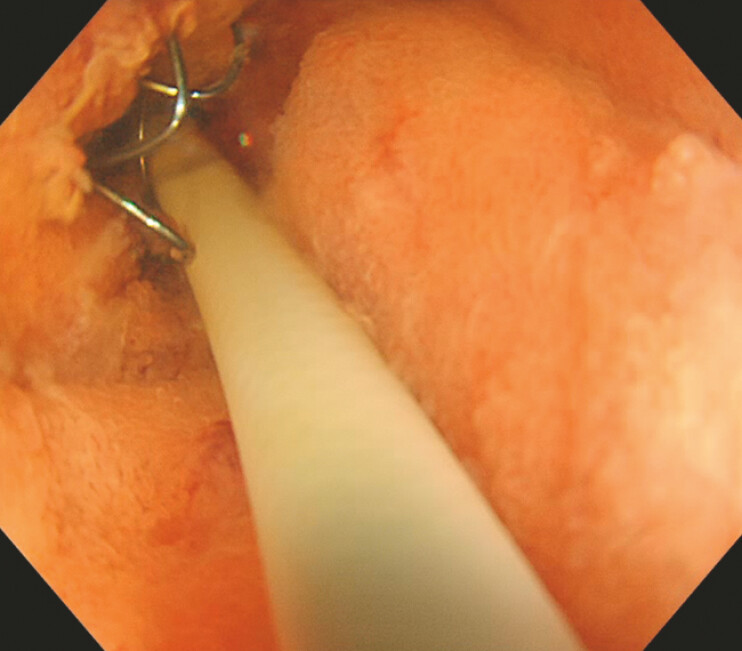
A cholangioscope was inserted through the stent.

**Fig. 4:**
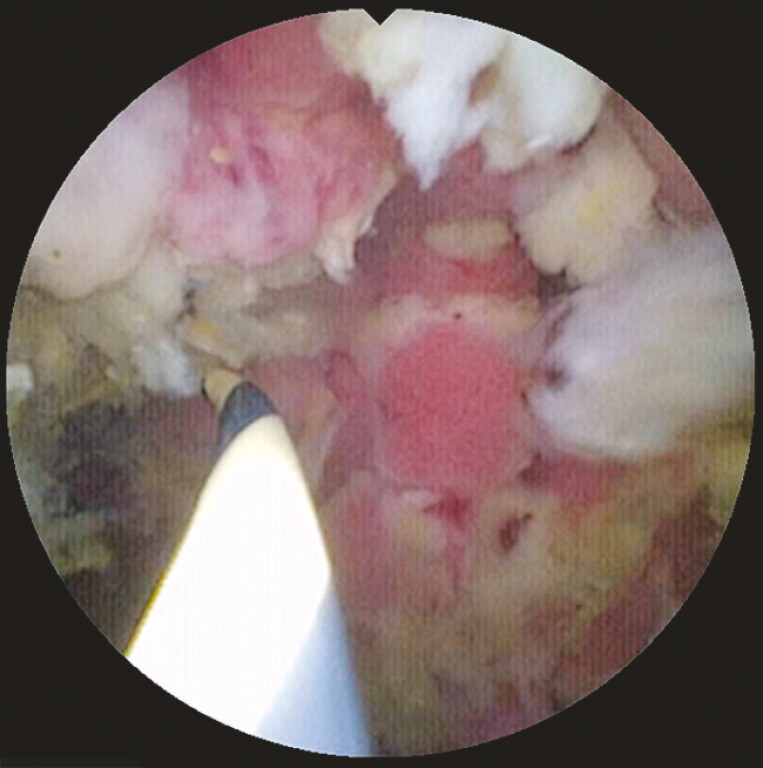
Under direct visualization, a guidewire was passed through the lumen of the stent and advanced into the distal duodenum.

**Fig. 5:**
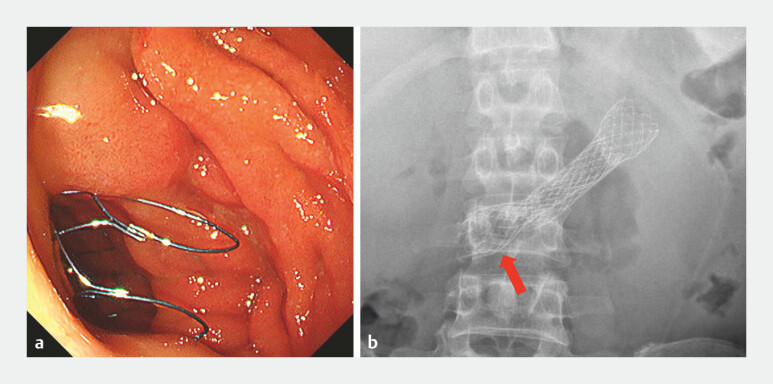
Well expansion of the proximal end of the second stent (red arrow), successfully overlapping the first stent. **a** An endoscopy view and **b** an abdominal X-ray view.

A cholangioscope was inserted through the stent ( Fig. 3 ). Under direct visualization, a guidewire was passed through the lumen of the stent, not through the stent mesh, and successfully advanced into the distal duodenum ( Fig. 4 , Video 1 ). A second 25 × 60 mm uncovered stent was successfully placed overlapping the first stent under the guidance of the guidewire ( Fig. 5 **a** ). An abdominal X-ray confirmed the complete expansion of the proximal end of the second stent ( Fig. 5 **b** ). The patient was able to eat semi-liquid foods without vomiting.

A novel cholangioscopic approach to overlapping stent placement in malignant duodenal horizontal segment obstruction.Video 1

To the best of our knowledge, this case represents the first successful attempt for direct cholangioscopic visualization without X-ray assisted during the placement of overlapping stents in malignant duodenal obstruction. The use of a cholangioscope for intraluminal visualization in technically challenging stent placement plays a crucial role by allowing for precise, radiation-free and safe deployment of overlapping stents.

Endoscopy_UCTN_Code_TTT_1AO_2AZ

